# SRGFormer: Semantic Role-Guided Graph Reasoning for Referring Remote Sensing Image Segmentation

**DOI:** 10.3390/s26144657

**Published:** 2026-07-22

**Authors:** Libang Liu, Jianxiang Li, Yaqin Li, Cao Yuan, Lili Fan, Xinyu Xiong, Wei Huang

**Affiliations:** 1 School of Mathematics and Computer Science, Wuhan Polytechnic University, Wuhan 430023, China; 20240712055@whpu.edu.cn (L.L.); lijianxiang@whpu.edu.cn (J.L.); leeyaqin@whpu.edu.cn (Y.L.); yc@whpu.edu.cn (C.Y.); fll12006@whpu.edu.cn (L.F.); 2 School of Computer Science and Engineering, Sun Yat-sen University, Guangzhou 510006, China; xiongxyowo@gmail.com

**Keywords:** referring remote sensing image segmentation, semantic role decomposition, relation-aware graph reasoning, vision–language segmentation, graph transformer, progressive mask refinement

## Abstract

Referring remote sensing image segmentation (RRSIS) aims to segment a target instance from remote sensing imagery according to a natural-language expression. It provides a flexible way to retrieve and localize specific objects in remote sensing scenes, benefiting intelligent Earth observation applications. Although existing methods have achieved promising progress by strengthening vision–language alignment, most of them still represent the expression as a holistic language feature and rely on convolution-dominated decoding for mask prediction. Such a paradigm tends to entangle target category, inter-object relation, and spatial position cues, making it difficult to distinguish the intended instance from multiple same-class distractors in complex remote sensing scenes. To address this limitation, we propose SRGFormer, a graph reasoning framework for RRSIS. Specifically, a semantic role decomposition (SRD) module decomposes the referring expression into target, relation, and position semantics, providing explicit linguistic priors for instance-level localization. Guided by the decomposed relation semantics, a semantic-relational graph transformer (SRGT) performs relation-aware graph reasoning over fused multi-scale visual features, enabling long-range dependency modeling among spatially distributed candidate instances. Furthermore, a progressive mask refinement (PMR) module continuously injects the decomposed semantic priors into semantic modulation, query initialization, and iterative mask decoding, thereby alleviating semantic fading during mask generation. Extensive experiments demonstrate that SRGFormer achieves substantial improvements on RefSegRS, attaining 66.08% mIoU and 76.93% oIoU (surpassing the prior state of the art by 3.96% and 2.83%, respectively) along with a notable 15.95% gain in Pr@0.7. Experiments on the additional RRSIS-D benchmark further demonstrate the general applicability of our approach, where SRGFormer maintains competitive performance (65.87% mIoU and 24.61% Pr@0.9) against existing methods. These results demonstrate that the proposed framework improves target localization and fine-grained mask prediction in complex remote sensing scenes.

## 1. Introduction

With the rapid development of deep learning for remote sensing data interpretation [1,2], intelligent Earth observation has achieved remarkable progress across a wide range of tasks, including image captioning [3], visual question answering [4], semantic segmentation [5,6,7], image recognition [8,9], and visual grounding [10,11,12]. Compared with conventional semantic segmentation and remote sensing instance segmentation [13], referring remote sensing image segmentation (RRSIS) provides a more flexible paradigm by allowing users to specify target objects through free-form natural language rather than predefined category labels [14,15]. Yuan et al. [16] first introduced RRSIS and constructed the RefSegRS benchmark, and Liu et al. [17] further proposed RMSIN together with the larger-scale RRSIS-D dataset [11,17]. This task requires fine-grained correspondence between linguistic descriptions and specific visual instances in aerial or satellite imagery. It is more challenging than category-level recognition because large-scale remote sensing scenes often contain numerous same-class distractors, weak object boundaries, substantial scale variation, and complex spatial layouts [11,12].

The language-guided retrieval capability enabled by RRSIS is particularly important for practical intelligent Earth observation applications, such as land use analysis [18,19], disaster response [20], military intelligence generation [21], environmental monitoring [22], and agricultural production management [23]. By allowing analysts to query and delineate particular objects with human-readable expressions, RRSIS expands the usability of remote sensing interpretation systems beyond fixed label sets. It also supports downstream scenarios such as geospatial question answering [4] and interactive scene understanding [10,12], where accurate object-level localization according to user intent is essential.

Recent RRSIS methods have achieved promising progress by introducing vision–language pre-training and Transformer-based cross-modal architectures [24,25,26]. Inspired by natural-image referring segmentation [14,27,28,29,30], existing RRSIS approaches usually strengthen vision–language interaction before mask prediction through pixel-word attention, language-guided multi-scale fusion, bidirectional interaction, or explicit cross-modal alignment [16,17,27,31,32,33,34,35,36]. These studies have demonstrated that enhancing cross-modal correspondence is essential for segmenting language-specified targets in complex aerial scenes.

**Figure 1:**
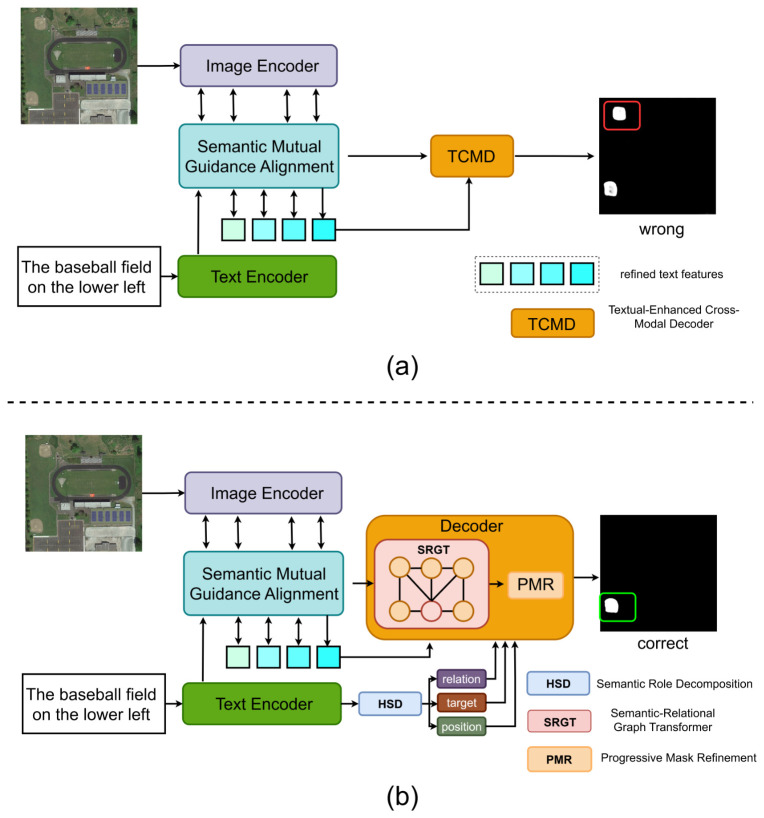
Motivation of SRGFormer. (**a**) Existing vision-language alignment methods use holistic language representations and ordinary decoders, resulting in mislocalization for relational/positional referring descriptions. (**b**) We separate referring expressions into target, relation and position semantics. The SRGT module executes relation-aware graph reasoning, and PMR embeds decomposed semantic cues iteratively to achieve accurate target localization in difficult remote sensing scenes.

However, we argue that a major bottleneck of RRSIS lies in decoder-side instance reasoning rather than encoder alignment. Existing methods, including CADFormer [32], represent the expression as a holistic embedding and mainly rely on local convolution for mask decoding. This design may entangle category, relational, and positional cues. We therefore retain the Swin–BERT encoder of CADFormer and focus on introducing semantic-role-guided graph reasoning into the decoder. As illustrated in Figure 1, an alignment-based model may correctly recognize the category *“baseball field”* but activate an instance that does not satisfy the positional constraint *“lower left”*. This example shows that successful RRSIS requires the decoder to preserve category, relational, and positional cues throughout mask prediction.

A major reason for this limitation is that most existing methods still feed the decoder with holistic language-conditioned visual features [16,17,27,32]. In such representations, target category, relational context, and spatial position are implicitly entangled within a unified feature space, providing limited explicit control over the visual interactions used for mask prediction [16,31,32]. Moreover, conventional convolution-dominated decoding mainly aggregates local neighborhoods, making it difficult to capture long-range dependencies among spatially separated candidate instances in large-scale remote sensing scenes [17,32,33]. Graph-structured reasoning provides a natural mechanism for modeling such long-range dependencies beyond fixed convolutional neighborhoods [30,37,38,39]. Nevertheless, directly applying conventional graph attention to RRSIS remains insufficient, because graph edges constructed only from visual similarity may reinforce visually similar distractors rather than the target instance specified by the expression [37,38]. Therefore, the central question of this work is: *how can decoder-side graph reasoning be explicitly guided by the semantic roles, especially the relation semantics, of the referring expression?*

To address this challenge, we propose SRGFormer, a semantic role-guided graph reasoning framework for referring remote sensing image segmentation. As shown in Figure 1, rather than redesigning the vision–language encoder, SRGFormer retains the established encoder-side cross-modal alignment paradigm and strengthens the decoder with structured semantic reasoning. Its core component, the semantic-relational graph transformer (SRGT), operates on fused multi-scale visual features using sparse multi-dilation neighborhoods and a global sink node. The graph attention logits are modulated by relation semantics, enabling long-range feature propagation to remain consistent with the relational cues expressed in the referring expression.

To provide structured linguistic guidance, the semantic role decomposition (SRD) module decomposes the expression into target, relation, and position embeddings. The relation embedding guides graph reasoning in SRGT, while the target and position embeddings provide complementary category and spatial constraints. The progressive mask refinement (PMR) module then reintroduces these semantic priors throughout iterative decoding to mitigate semantic fading. Together, SRD, SRGT, and PMR form a unified reasoning pipeline that converts structured language cues into accurate instance localization and pixel-level segmentation.

The main contributions of this work are summarized as follows:

- We propose SRGFormer, a semantic role-guided graph reasoning framework for RRSIS, which introduces semantic role decomposition and relation-aware graph reasoning into the decoding stage to enhance instance-level discrimination in complex remote sensing scenes.
- We design a unified reasoning pipeline composed of SRD, SRGT, and PMR. It decomposes referring expressions into target, relation, and position semantics, performs relation-conditioned graph reasoning, and progressively injects semantic priors into mask refinement to alleviate semantic fading.
- We evaluate SRGFormer on the RefSegRS and RRSIS-D benchmarks. It achieves state-of-the-art performance on RefSegRS, with 66.08% mIoU and 76.93% oIoU, and maintains strong performance on RRSIS-D.

## 2. Related Work

### 2.1. Referring Image Segmentation

Referring image segmentation (RIS) aims to segment a target object in a natural image according to a free-form linguistic expression [14]. Early RIS methods mainly relied on fully convolutional networks and recurrent language encoders to fuse global sentence vectors with visual features [14,40,41], but they struggled to establish fine-grained word–region correspondence. With the development of vision–language pre-training and Transformer architectures [24,25], recent methods increasingly perform token-level cross-modal interaction. LAVT [27] integrates language-aware vision encoding and decoding through multi-head cross-attention between text tokens and multi-scale visual features, achieving strong performance on general referring segmentation benchmarks. CMSA [42], BRINet [43], and VLT [29] further leverage cross-modal self/interactive attention to deeply fuse language and visual features. Subsequent works further explore query-based decoding, multi-stage refinement, and explicit linguistic reasoning to improve instance-level grounding under complex expressions [29,30,44]. These studies demonstrate that effective RIS requires not only category recognition, but also disentangled understanding of relational and spatial cues in language. However, most general-domain methods are designed for natural images with relatively compact scenes and salient objects, and their holistic language fusion strategies are not directly sufficient for large-scale remote sensing scenarios with weak boundaries and dense distractors.

### 2.2. Referring Remote Sensing Image Segmentation

Referring remote sensing image segmentation (RRSIS) extends RIS to aerial and satellite imagery [16]. Compared with natural images, remote sensing scenes are characterized by large coverage, significant scale variation, weak target boundaries, and frequently occurring same-class instances, making instance discrimination substantially more challenging. Yuan et al. [16] first introduced RRSIS into the remote sensing domain and constructed the RefSegRS benchmark [15,16]. LGCE [16] enhances multi-scale visual representations with language guidance on this dataset. RMSIN [17] further introduces an oriented-aware decoder to handle direction-sensitive objects and establishes a widely used protocol on RRSIS-D [11,17]. More recently, CADFormer [32] designs bidirectional vision–language alignment in the encoder and a textual-enhanced cross-modal decoder (TCMD) for progressive, language-aware mask prediction, achieving strong results on RefSegRS and RRSIS-D. CroBIM [33] and DANet [34] further explore bidirectional vision–language interaction and explicit dual alignment for RRSIS. RSAM [35] extends SAM2 with a two-way guidance module to mutually refine vision and language features and constructs the large-scale RISORS benchmark. MPBF [36] introduces prompt-guided bidirectional fusion for parameter-efficient cross-modal alignment. FIANet [31] also explores fine-grained image–text alignment for remote sensing referring segmentation. Despite their improved cross-modal alignment, most existing methods still represent the expression as a unified language context and rely primarily on convolutional decoding. Consequently, they offer limited control over category, relational, and positional cues when multiple same-class candidates coexist. SRGFormer addresses this limitation by decomposing BERT [25] token features into role-specific embeddings and using them to guide graph-based decoding.

### 2.3. Graph-Based Visual Reasoning and Fusion

Graph modeling provides a flexible way to encode structural dependencies among visual entities beyond fixed convolutional neighborhoods. Graph convolutional networks and graph attention networks (GATs) [37] aggregate context from adaptive neighbors and have been widely used in semantic segmentation, scene parsing, and relational reasoning. In natural image understanding, graph structures are often built over objects, superpixels, or region proposals to propagate relational cues among distant entities [38,39]. In remote sensing analysis, graph-based methods have also been explored for land-cover classification, change detection, and object relationship modeling, where long-range dependencies and irregular object distributions are common. For vision–language tasks, graph reasoning is further used to connect linguistic concepts with visual nodes, enabling structured cross-modal interaction beyond simple feature concatenation or global pooling [30,38]. Nevertheless, existing RRSIS methods still rely predominantly on attention-based or convolution-based fusion, and rarely introduce explicit graph reasoning on fused multi-scale feature maps under relation-specific language control. This limits their ability to discriminate among spatially distant but relationally coupled instances in large remote sensing scenes. Different from prior graph segmentation methods that use category-agnostic message passing, our semantic-relational graph transformer (SRGT) constructs relation-constrained local graph attention on fused visual features, where relational semantics dynamically modulate attention logits across multi-dilation neighborhoods and a global sink node. In this way, SRGT enlarges the effective receptive field and injects language-guided relational priors into visual reasoning, complementing the early semantic parsing of SRD and the progressive constraint propagation of PMR in SRGFormer.

## 3. Methodology

### 3.1. Overall Framework

**Figure 2:**
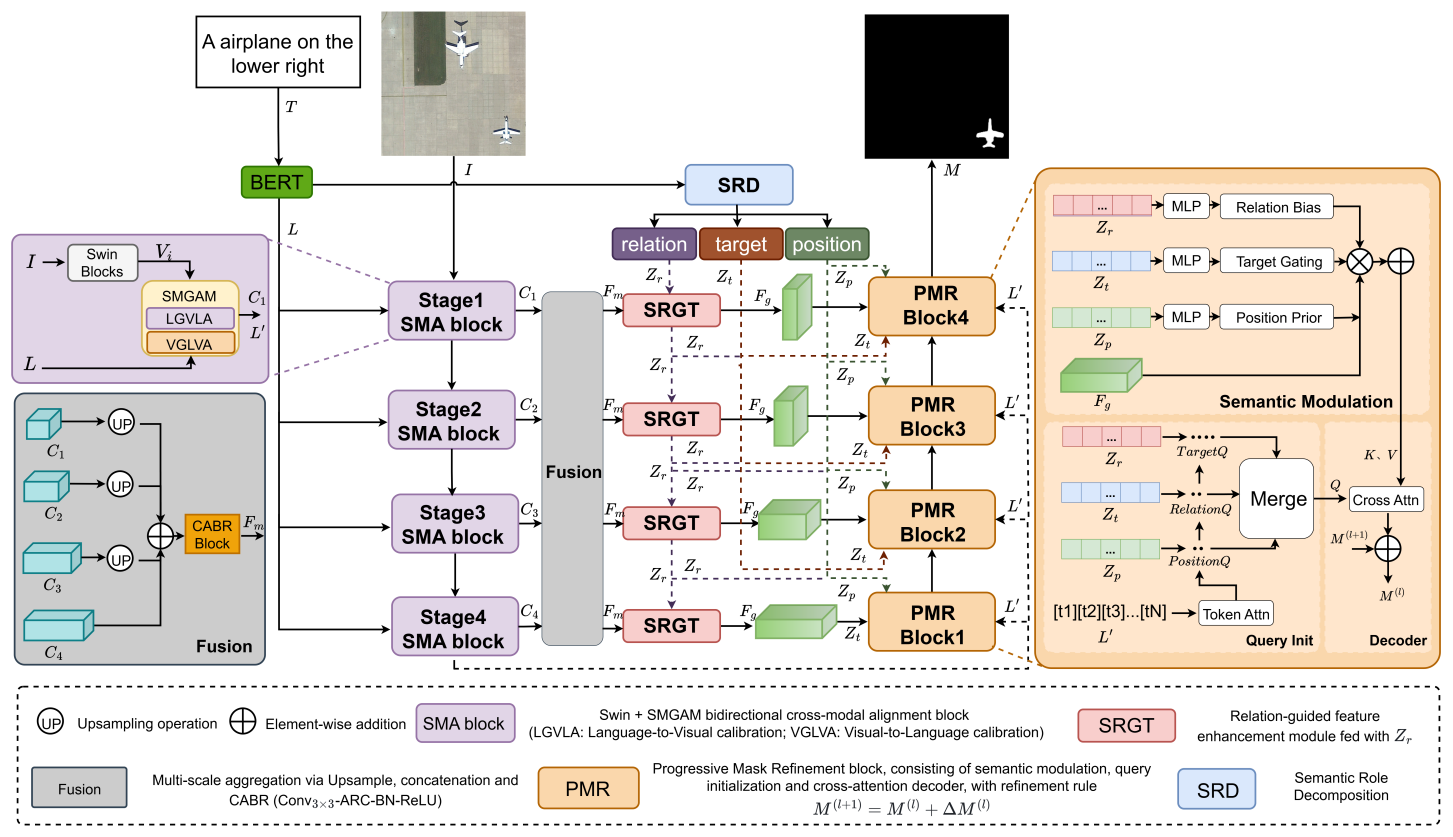
Overall architecture of the proposed SRGFormer. The framework consists of four main components: stage-wise mutual alignment encoder, semantic role decomposition (SRD), semantic-relational graph transformer (SRGT), and progressive mask refinement (PMR).

Given a remote sensing image *I* and a referring expression *T*, SRGFormer follows the encoder–decoder architecture shown in Figure 2. It comprises a stage-wise mutual alignment encoder, SRD, SRGT, and PMR. The Swin Transformer [26] extracts hierarchical visual features from *I*, while BERT [25] encodes *T* into token-level features *L*. SRD then generates role-specific language priors that guide graph reasoning and progressive mask prediction.

**Figure 3:**
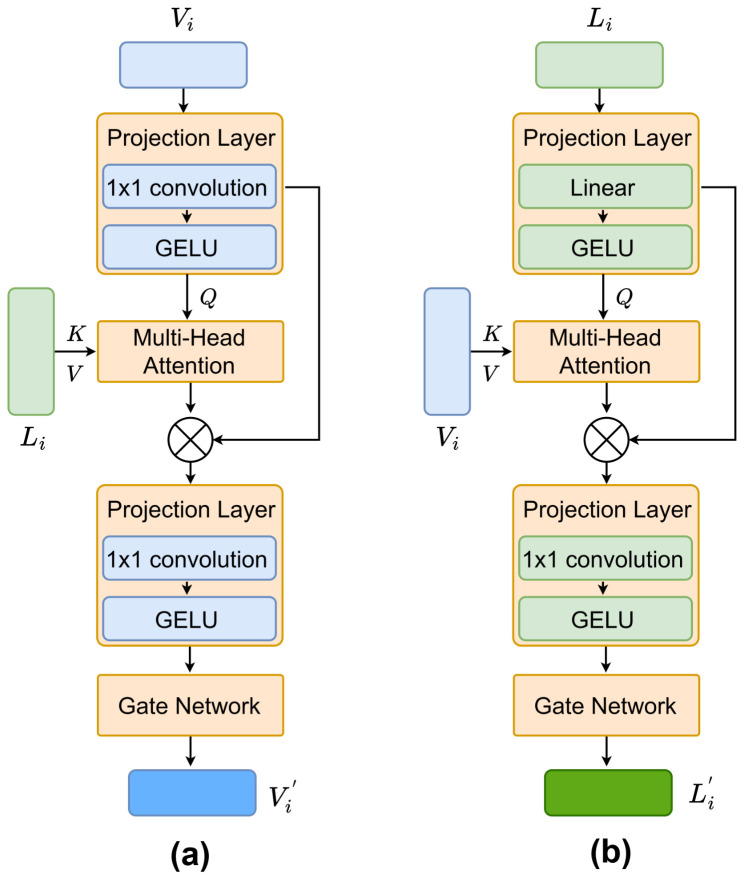
Structure of the Semantic Mutual Guidance Alignment Module (SMGAM). (**a**) Language-guided visual alignment. (**b**) Vision-guided language alignment.

As shown in the left part of Figure 2, we adopt a stage-wise vision–language alignment encoder to obtain multi-scale cross-modal representations. Following the strong encoder-side alignment design of CADFormer [32], each encoder stage is formulated as a Stage-wise Mutual Alignment (SMA) block, which contains Swin Transformer blocks and the Semantic Mutual Guidance Alignment Module (SMGAM). The detailed structure of SMGAM is shown in Figure 3. It consists of two complementary submodules: the Language-Guided Vision–Language Alignment (LGVLA) module and the Vision-Guided Language–Vision Alignment (VGLVA) module. Given the visual feature $V_{i}$ and the stage-specific language feature $L_{i}$, LGVLA uses language features as guidance to calibrate visual representations, while VGLVA uses visual features to refine language representations. Through such bidirectional interaction, each SMA block produces an enhanced visual feature $C_{i}$ and an updated language feature $L_{i}^{\prime }$. After four-stage alignment, the encoder outputs multi-scale visual features ${\left\{C_{i}\right\}}_{i=1}^{4}$ and the final vision-aligned language feature $L^{\prime }$.

In parallel with the stage-wise cross-modal alignment, the BERT language feature *L* is also fed into the proposed semantic role decomposition (SRD) module. As shown in the upper middle part of Figure 2, SRD decomposes the referring expression into three role-specific semantic embeddings: target semantics $z_{t}$, relation semantics $z_{r}$, and position semantics $z_{p}$. These three semantics correspond to three essential questions in referring segmentation: *what* object should be segmented, *which* instance should be selected, and *where* the target is located. Among them, the relation semantic embedding $z_{r}$ plays a central role in guiding graph-based relational reasoning, while $z_{t}$ and $z_{p}$ provide category-level and spatial constraints for the subsequent mask refinement process.

The multi-scale encoder features ${\left\{C_{i}\right\}}_{i=1}^{4}$ are then aggregated by the Fusion module. Specifically, feature maps from different stages are first projected to a unified channel dimension and spatially aligned by upsampling. The aligned features are then concatenated and processed by a convolutional aggregation block, denoted as CABR, to generate the fused representation $F_{m}$. This fused feature preserves both high-level semantic context and low-level spatial details, providing a unified visual basis for the following relation-aware reasoning stage.

Although the SMA encoder establishes fine-grained vision–language correspondence, its output features are still mainly decoded through local feature propagation if no additional reasoning mechanism is introduced. This is insufficient for RRSIS, because the referred target is often identified by long-range relations with other objects rather than by local appearance alone. To address this limitation, we introduce the semantic-relational graph transformer (SRGT) as the core reasoning component of SRGFormer. As shown in the middle part of Figure 2, SRGT takes the fused visual representation $F_{m}$ as input and performs graph-based feature interaction under the guidance of the relation semantic prior $z_{r}$. Instead of relying on unconstrained visual similarity, SRGT injects $z_{r}$ into graph attention to encourage message passing along relation-consistent visual regions. In this way, SRGT produces a relation-enhanced feature representation $F_{g}$, which strengthens long-range instance discrimination in complex remote sensing scenes.

Finally, the relation-enhanced features are progressively decoded by the progressive mask refinement (PMR) module. As illustrated in the right part of Figure 2, each PMR block consists of semantic modulation, semantic-aware query initialization, and a cross-attention-based refinement decoder. In the semantic modulation branch, $z_{t}$, $z_{r}$, and $z_{p}$ are transformed into target gating, relation bias, and position prior, respectively, and are jointly used to modulate the graph-enhanced feature $F_{g}$. In the query initialization branch, the three decomposed semantic embeddings initialize target-, relation-, and position-aware queries, which are further enriched by the aligned language feature $L^{\prime }$ through token attention. These semantic-aware queries interact with the visual memory through cross-attention and predict residual mask updates. The mask is therefore refined in a progressive manner:

(1) $$M^{\left(l+1\right)}=M^{\left(l\right)}+\Delta M^{\left(l\right)},$$

where $M^{\left(l\right)}$ denotes the mask prediction at the *l*-th refinement stage and $\Delta M^{\left(l\right)}$ is the residual update predicted by the corresponding PMR block. Through this iterative process, SRGFormer maintains explicit semantic constraints throughout decoding and finally outputs the segmentation mask *M*.

### 3.2. Semantic Role Decomposition (SRD)

Referring expressions in remote sensing imagery usually contain multiple types of linguistic cues that contribute differently to target localization [31,38]. For example, in the expression “the airplane on the lower right”, the word “airplane” indicates the target category, while “lower right” provides spatial guidance for selecting the intended instance. In more complex scenes, relational words such as “near”, “beside”, or “between” are also crucial for distinguishing the referred object from surrounding same-category candidates [39,43]. Existing RRSIS methods usually encode the entire expression into a holistic language representation derived from BERT [25] and inject it into visual features through cross-modal attention [16,27,32]. Although such holistic conditioning is effective for simple expressions, it entangles category, relation, and position cues within a single embedding space, making it difficult for the decoder to selectively exploit different linguistic roles during instance reasoning [17,31].

**Figure 4:**
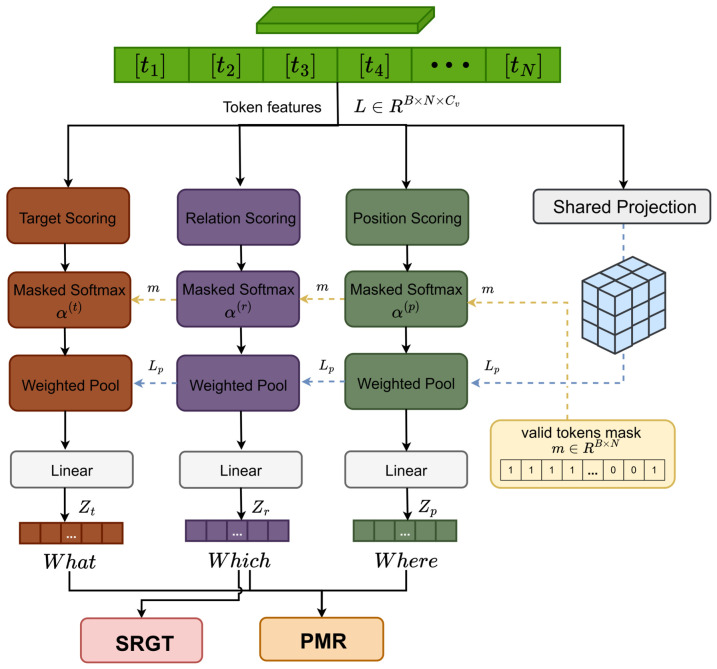
Structure of the proposed semantic role decomposition (SRD) module, which extracts target, relation, and position semantic representations from language features.

To provide explicit semantic priors for relation-aware decoding, we propose a lightweight semantic role decomposition (SRD) module, as illustrated in Figure 4. SRD decomposes the token-level language feature into three role-specific semantic embeddings: target semantics $z_{t}$, relation semantics $z_{r}$, and position semantics $z_{p}$. These three embeddings correspond to the three key questions in referring segmentation [14]: *what* object should be segmented, *which* instance should be selected, and *where* the target is located. The decomposed semantics are subsequently used by different components of the decoder: $z_{r}$ provides the relation prior for the semantic-relational graph Transformer (SRGT), while $\left\{z_{t},z_{r},z_{p}\right\}$ jointly guide the progressive mask refinement (PMR) module.

Given the token-level language feature extracted by BERT,

(2) $$L\in R^{B\times N\times C_{l}},$$

where *B*, *N*, and $C_{l}$ denote the batch size, token number, and language feature dimension, respectively, SRD contains two parallel paths: a role-aware scoring path and a shared projection path.

First, the shared projection path maps the original language feature into the decoder embedding space:

(3) $$L_{p}=\mathrm{SharedLinear}\left(L\right)\in R^{B\times N\times C_{v}},$$

where $C_{v}$ denotes the decoder feature dimension. This projection ensures dimensional compatibility between the decomposed language semantics and the subsequent visual decoding features. The projected feature $L_{p}$ is used as the value feature for semantic aggregation.

Second, the role-aware scoring path estimates token importance from three semantic perspectives. Since different semantic roles usually correspond to different subsets of words, SRD employs three independent scoring branches:

(4) $$S_{k}={\mathrm{Linear}}_{k}\left(L\right)\in R^{B\times N},\;k\in \left\{t,r,p\right\},$$

where *t*, *r*, and *p* denote target, relation, and position semantics, respectively. The three branches are designed to aggregate different types of linguistic cues, providing target, relation, and position semantic representations for subsequent decoding.

To suppress padded or invalid tokens, the valid-token mask $m\in {\left\{0,1\right\}}^{B\times N}$ is applied before normalization. The role-specific token attention weights are computed as

(5) $$\alpha^{\left(k\right)}=\mathrm{Softmax}\left(S_{k}+\left(1-m\right)\cdot \left(-10^{4}\right)\right),\;k\in \left\{t,r,p\right\}.$$

In this way, only valid tokens participate in semantic role aggregation.

The obtained attention weights are then used to perform weighted pooling over the projected token feature $L_{p}$:

(6) $${\tilde{z}}_{k}=\sum_{i=1}^{N}\alpha_{i}^{\left(k\right)}L_{p}\left(i\right),\;k\in \left\{t,r,p\right\}.$$

Finally, three dedicated output projections generate the role-specific semantic embeddings:

(7) $$z_{k}={\mathrm{Linear}}_{k}^{out}\left({\tilde{z}}_{k}\right)\in R^{B\times C_{v}},\;k\in \left\{t,r,p\right\}.$$

The resulting embeddings have distinct functions in the proposed framework. The target semantic embedding $z_{t}$ describes the object category and is used to enhance category-relevant visual responses. The relation semantic embedding $z_{r}$ captures instance-discriminative relational cues and is used as the key linguistic prior for SRGT. By injecting $z_{r}$ into graph attention, SRGT performs relation-conditioned message passing rather than appearance-only feature propagation. The position semantic embedding $z_{p}$ provides coarse spatial constraints and helps PMR narrow the search region during mask refinement.

SRD requires no semantic-role annotations and is trained end-to-end using only segmentation supervision. The relation embedding $z_{r}$ guides SRGT, whereas all three embeddings constrain PMR. SRD therefore converts holistic language conditioning into role-specific priors for subsequent decoding.

### 3.3. Semantic-Relational Graph Transformer (SRGT)

Although the SMA encoder establishes fine-grained vision–language correspondence [27,32], the subsequent mask decoding process still requires effective relational reasoning among spatially distributed objects. This is particularly important in referring remote sensing image segmentation, where the target is often specified by relational or positional descriptions, such as *“the building beside the stadium”* or *“the ship near the harbor”* [11,17]. In such cases, accurate localization cannot be achieved by local appearance modeling alone, because the decoder needs to distinguish the referred instance from other visually similar candidates according to the relation expressed in language [31,32].

**Figure 5:**
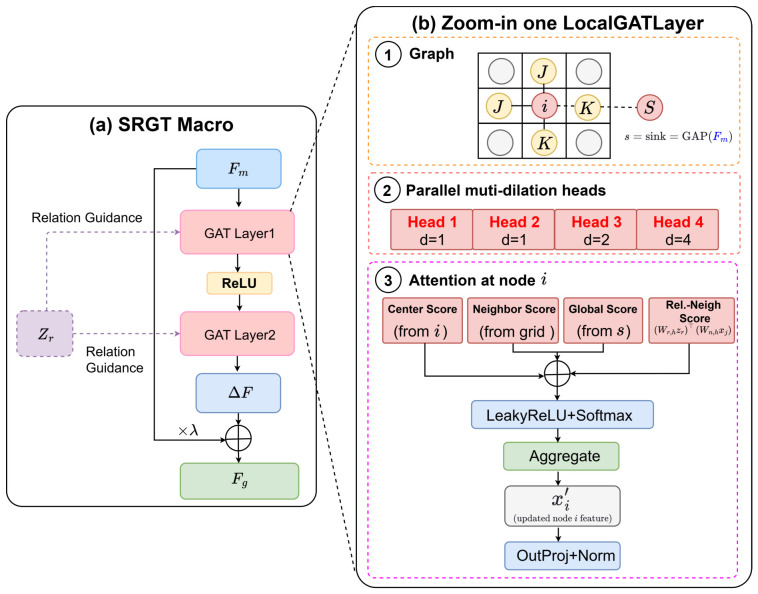
Structure of the proposed semantic-relational graph transformer (SRGT). (**a**) Overall architecture. (**b**) Relation-aware graph attention with multi-dilation neighborhoods and a global sink node.

Dense global self-attention incurs substantial computational cost when applied to high-resolution remote sensing features [24]. Moreover, conventional self-attention and graph attention derive interaction weights primarily from visual similarity [37,38] and may therefore overlook the relational constraints expressed in the referring query. As illustrated in Figure 5, SRGT addresses these limitations by constructing a sparse graph and using the relation embedding $z_{r}$ to modulate the attention logits. Consequently, message passing is jointly determined by visual compatibility and the relational semantics of the expression [30,39].

#### 3.3.1. Multi-Scale Feature Fusion

Before graph reasoning, the multi-scale features produced by the SMA encoder are first aggregated into a unified representation. Specifically, the four-stage encoder outputs ${\left\{C_{i}\right\}}_{i=1}^{4}$ are projected to a common channel dimension and aligned to the spatial resolution of $C_{1}$. The aligned features are concatenated and fused by a convolutional aggregation block:

(8) $$\begin{array}{cc} F_{m} & =\mathrm{ReLU}(\mathrm{BN}(\mathrm{ARC}({\mathrm{Conv}}_{3\times 3}(\mathrm{Concat}[\mathrm{Up}\left(\mathrm{Proj}\left(C_{4}\right)\right),\mathrm{Up}\left(\mathrm{Proj}\left(C_{3}\right)\right),\\ & \;\;\mathrm{Up}\left(\mathrm{Proj}\left(C_{2}\right)\right),\mathrm{Proj}\left(C_{1}\right)]))))\\ & \in R^{B\times C_{v}\times h\times w}. \end{array}$$

where Proj(·) denotes channel projection, Up(·) denotes bilinear upsampling, and ARC(·) denotes adaptive rotated convolution. The fused feature $F_{m}$ preserves both high-level semantic context and low-level spatial details, and serves as the input of SRGT.

#### 3.3.2. Macro Structure of SRGT

As shown in Figure 5a, SRGT consists of two stacked LocalGAT layers with a nonlinear activation in between. Given the fused feature map $F_{m}$ and the relation semantic embedding $z_{r}$, SRGT predicts a relation-enhanced residual feature:

(9) $$\Delta F={\mathrm{LocalGAT}}_{2}\left(\mathrm{ReLU}\left({\mathrm{LocalGAT}}_{1}\left(F_{m},z_{r}\right)\right),z_{r}\right).$$

The final graph-enhanced feature is obtained through a residual connection:

(10) $$F_{g}=F_{m}+\lambda \Delta F,$$

where λ is a learnable scaling factor initialized to 0.1. This residual formulation preserves the original fused visual representation while gradually incorporating relation-aware graph information. The learnable scale also stabilizes early training and prevents graph reasoning from overwhelming the original visual features.

#### 3.3.3. Local Graph Construction with Multi-Dilation Heads

Each spatial position in $F_{m}$ is regarded as a graph node. Let $x_{i}\in R^{C_{v}}$ denote the feature of node *i*. Instead of constructing a fully connected graph over all spatial locations, each LocalGAT layer builds sparse neighborhoods around each node using multi-dilated 3×3 windows. For a dilation rate *d*, the neighborhood of node *i* is denoted as $N_{d}\left(i\right)$. As illustrated in Figure 5b, different attention heads use different dilation rates, e.g., {1,1,2,4}, so that SRGT can jointly capture short-range context and long-range object relations.

To further enable efficient global context exchange, a global sink node *s* is introduced:

(11) $$x_{s}=\mathrm{GAP}\left(F_{m}\right),$$

where GAP(·) denotes global average pooling. The sink node acts as a compact scene-level memory. It collects global contextual information and participates in the attention computation of every node, enabling long-range information interaction without dense global attention.

#### 3.3.4. Relation-Aware Graph Attention

The core of SRGT is relation-aware graph attention. For each node *i*, the attention score is computed by combining the center-node compatibility, neighboring-node compatibility, global sink interaction, and edge-dependent relation–visual compatibility derived from $z_{r}$. Unlike a shared scalar bias, the relation-aware compatibility varies with different visual nodes and therefore directly influences the relative attention distribution. For the *h*-th head with dilation rate $d_{h}$, the unnormalized attention logit between node *i* and its neighboring node *j* is defined as

(12) $$e_{ij}^{\left(h\right)}=a_{q,h}^{⊤}W_{q,h}x_{i}+a_{k,h}^{⊤}W_{k,h}x_{j}+{\left(W_{r,h}z_{r}\right)}^{⊤}\left(W_{n,h}x_{j}\right),\;j\in N_{d_{h}}\left(i\right).$$

where $W_{q,h}$ and $W_{k,h}$ are visual projections for the center node and neighboring node, respectively. The third term represents the relation–visual compatibility between the relation semantic embedding $z_{r}$ and the neighboring visual node. Different from a neighborhood-shared scalar bias, this formulation depends on the specific neighboring node *j* and therefore provides edge-dependent relation guidance before softmax normalization. The corresponding relation guidance mechanism is illustrated in Figure 5b, where the relation semantic embedding interacts with visual nodes to generate relation-aware compatibility scores.

Similarly, the attention logit between node *i* and the global sink node *s* is computed as

(13) $$e_{is}^{\left(h\right)}=a_{q,h}^{⊤}W_{q,h}x_{i}+a_{s,h}^{⊤}W_{s,h}x_{s}+{\left(W_{r,h}z_{r}\right)}^{⊤}\left(W_{g,h}x_{s}\right),$$

where $W_{s,h}$ and $W_{g,h}$ denote the visual and relation-aware projections for the global sink node, respectively. This formulation enables the global context interaction to be dynamically guided by the relation semantics. The attention weights are then normalized over the union of local graph neighbors and the global sink node:

(14) $$\alpha_{ij}^{\left(h\right)}=\frac{\exp\left(\mathrm{LeakyReLU}(e_{ij}^{\left(h\right)})\right)}{\sum_{k\in N_{d_{h}}\left(i\right)\cup \left\{s\right\}}\exp\left(\mathrm{LeakyReLU}(e_{ik}^{\left(h\right)})\right)},\;j\in N_{d_{h}}\left(i\right)\cup \left\{s\right\}.$$

The updated feature of node *i* in the *h*-th head is obtained by relation-aware message aggregation:

(15) $${\hat{x}}_{i}^{\left(h\right)}=\sum_{j\in N_{d_{h}}\left(i\right)\cup \left\{s\right\}}\alpha_{ij}^{\left(h\right)}W_{h}x_{j}.$$

The outputs of all heads are concatenated and projected to obtain the updated node representation:

(16) $$x_{i}^{\prime }=\mathrm{Norm}\left(\mathrm{OutProj}\left({\mathrm{Concat}}_{h=1}^{H}{\hat{x}}_{i}^{\left(h\right)}\right)\right).$$

SRGT combines efficient and language-conditioned visual reasoning. The multi-dilation neighborhoods capture context at different spatial ranges, the sink node provides scene-level information, and $z_{r}$ aligns graph propagation with the relational cues in the expression. The resulting feature $F_{g}$ is then passed to PMR.

### 3.4. Progressive Mask Refinement (PMR)

After SRGT, the fused visual representation is enhanced with relation-aware graph reasoning. However, translating such high-level relational features into accurate pixel-level masks remains non-trivial. In many referring segmentation decoders, language guidance is mainly injected at early fusion stages [16,27,32], while the subsequent mask generation process is dominated by visual feature propagation. As a result, the linguistic constraints may gradually fade during decoding [30,44], leading to ambiguous localization when multiple visually similar instances coexist in remote sensing scenes [17,31].

To alleviate this issue, we propose a progressive mask refinement (PMR) module, as shown in the right part of Figure 2. PMR aims to continuously inject decomposed semantic priors into mask decoding rather than using language guidance only once [28,29,40]. Specifically, each PMR block consists of three parts: semantic prior modulation, semantic-aware query initialization, and cross-attention-based residual mask refinement. Given the graph-enhanced feature $F_{g}$, the decomposed semantic embeddings $\left\{z_{t},z_{r},z_{p}\right\}$, and the vision-aligned language feature $L^{\prime }$, PMR progressively refines the segmentation mask from coarse localization to fine-grained prediction.

#### 3.4.1. Semantic Prior Modulation

The three decomposed semantic roles contribute differently to target localization. Target semantics indicate category-related responses, relation semantics distinguish the referred instance from surrounding candidates, and position semantics provide coarse spatial constraints. Therefore, instead of simply concatenating them, PMR converts $z_{t}$, $z_{r}$, and $z_{p}$ into three complementary modulation priors, as illustrated in the semantic modulation branch of Figure 2.

First, the target semantic embedding $z_{t}$ is transformed into a channel-wise target gate:

(17) $$g_{t}=\sigma \left({\mathrm{MLP}}_{t}\left(z_{t}\right)\right),$$

where $g_{t}\in R^{B\times C_{v}\times 1\times 1}$ and σ(·) denotes the sigmoid function. The target gate adaptively enhances category-relevant channels and suppresses irrelevant visual responses.

Second, the relation semantic embedding $z_{r}$ is mapped into a relation bias:

(18) $$b_{r}={\mathrm{MLP}}_{r}\left(z_{r}\right),$$

where $b_{r}\in R^{B\times C_{v}\times 1\times 1}$. This bias further emphasizes relation-consistent feature responses, which is beneficial for discriminating the referred target from visually similar distractors.

Third, the position semantic embedding $z_{p}$ is used to generate a soft spatial prior:

(19) $$P=\sigma \left(\sum_{k=1}^{4}\gamma_{k}\left(z_{p}\right)\Phi_{k}\left(x,y\right)\right),$$

where $P\in R^{B\times 1\times h\times w}$, and

(20) Φ(x,y)=[x,y,xy,1]

denotes a fixed coordinate basis. Compared with explicit coordinate regression, this formulation provides flexible spatial guidance while preserving the ability to adapt to targets of different shapes and scales.

The three semantic priors are then applied to the graph-enhanced feature $F_{g}$:

(21) $$\hat{F}=\left(F_{g}⊙\left(1+g_{t}\right)+b_{r}\right)⊙\left(1+P\right),$$

where ⊙ denotes element-wise multiplication. This modulation process follows a semantic-to-spatial constraint strategy: target gating first enhances category-relevant responses, relation bias then strengthens relation-consistent regions, and position modulation finally narrows the search space to spatially plausible areas.

#### 3.4.2. Semantic-Aware Query Initialization

As shown in the query initialization branch of Figure 2, PMR further constructs semantic-aware queries from the decomposed language priors. Conventional Transformer decoders usually use learnable object queries that are independent of the referring expression. Such queries are effective for generic segmentation but provide limited explicit guidance for referring segmentation, where the target instance is determined by the input expression.

To introduce stronger linguistic awareness, PMR initializes three groups of queries from $z_{t}$, $z_{r}$, and $z_{p}$, corresponding to target-oriented, relation-oriented, and position-oriented queries:

(22) $$Q_{t}={\mathrm{MLP}}_{qt}\left(z_{t}\right),\;Q_{r}={\mathrm{MLP}}_{qr}\left(z_{r}\right),\;Q_{p}={\mathrm{MLP}}_{qp}\left(z_{p}\right).$$

The three query groups are then merged to form the initial semantic query set:

(23) $$Q^{\left(0\right)}=\mathrm{Merge}\left(Q_{t},Q_{r},Q_{p}\right).$$

Since semantic decomposition may compress some fine-grained token-level information, the aligned language feature $L^{\prime }$ generated by the SMA encoder is further used to enrich the query representation through token attention:

(24) $$Q^{\left(0\right)}=\mathrm{TokenAttn}\left(Q^{\left(0\right)},L^{\prime }\right).$$

In this way, PMR combines structured semantic priors from SRD with detailed token-level information preserved by cross-modal alignment.

#### 3.4.3. Progressive Residual Mask Refinement

Instead of predicting the final segmentation mask in a single step, PMR adopts a progressive refinement strategy. A coarse mask prediction is first generated from the semantically modulated feature:

(25) $$M^{\left(0\right)}={\mathrm{Head}}_{base}\left(\hat{F}\right)+\mathrm{Concat}\left(0,P\right),$$

where the background channel receives zero bias and the foreground channel is initialized with the position prior *P*. This provides a spatially informed starting point for subsequent refinement.

The visual memory is obtained by flattening the modulated feature map:

(26) $$V=\mathrm{Flatten}(\hat{F}).$$

At the *l*-th refinement stage, the semantic-aware queries interact with the visual memory through a cross-attention decoder:

(27) $$Q^{\left(l+1\right)}=\mathrm{DecoderLayer}\left(Q^{\left(l\right)},V\right).$$

The updated queries are then used to predict a residual mask update:

(28) $$M^{\left(l+1\right)}=M^{\left(l\right)}+\Delta M^{\left(l\right)}\left(Q^{\left(l+1\right)},\hat{F}\right),$$

where $\Delta M^{\left(l\right)}$ is produced by a lightweight refinement head. This residual formulation allows the model to gradually correct localization errors and refine object boundaries.

To prevent semantic fading during iterative decoding, each refinement stage is additionally conditioned on a global semantic context:

(29) $$z_{avg}=\frac{z_{t}+z_{r}+z_{p}}{3}.$$

The semantic context is injected into the refinement head together with the query representation, ensuring that target, relation, and position cues remain active throughout mask generation. Consequently, PMR converts the relation-enhanced feature $F_{g}$ into the final segmentation mask *M* through persistent semantic modulation and progressive residual refinement.

### 3.5. Loss Function

The overall training objective combines segmentation fidelity, deep supervision, and boundary-aware regularization:

(30) $$L=L_{ds}+\lambda_{bd}L_{bd},$$

where $\lambda_{bd}=0.5$. The base segmentation loss is

(31) $$L_{seg}=\left(1-\beta \right)L_{CE}+\beta L_{Dice},$$

with β=0.1. Deep supervision is applied to all PMR stages:

(32) $$L_{ds}=\sum_{l=0}^{L}w_{l}\;L_{seg}\left(M^{\left(l\right)},Y\right),$$

where stage weights are set to [0.3,0.3,0.5,1.0]. Boundary loss is defined as

(33) $$L_{bd}={∥B\left(M^{\left(L\right)}\right)-B\left(Y\right)∥}_{1},$$

where B(·) extracts soft boundaries via max-pooling based dilation/erosion with kernel size 3.

## 4. Experiments

### 4.1. Datasets

We conduct experiments on two public RRSIS benchmarks, including RefSegRS [16] and RRSIS-D [17]. The detailed statistics of the two datasets are summarized in Table 1.

RefSegRS. RefSegRS is the first benchmark proposed for referring remote sensing image segmentation [16]. It is constructed on top of the SkyScapes dataset [15], which consists of high-resolution aerial remote sensing imagery collected from airborne platforms. The dataset contains 4420 image–expression–mask triplets across 285 scenes and provides diverse urban scenarios for evaluating language-guided object localization and segmentation. Following the official split, we use 2172 triplets from 151 scenes for training, 431 triplets from 31 scenes for validation, and 1817 triplets from 103 scenes for testing, with no scene overlap among the three subsets. The expressions describe object categories, attributes, and spatial relations, and the dataset is characterized by large scale variation and many small or scattered ground objects.

RRSIS-D. The RRSIS-D dataset is constructed based on DIOR-RSVG and the remote sensing visual grounding dataset RSVG [11]. It contains high-resolution overhead remote sensing imagery with more than 17,402 image–expression–mask triplets covering 20 object categories, providing a larger-scale benchmark for evaluating referring expression-guided segmentation in complex remote sensing scenes. Following the standard split in prior works [17,32], we use 12,181 samples for training, 1740 for validation, and 3481 for testing. The average expression length is about 6.8 words. Although RRSIS-D is currently the most widely used RRSIS benchmark, its target objects are often relatively salient in large scenes.

### 4.2. Evaluation Metrics

Following previous works [16,17,27], we adopt three widely used evaluation metrics: mean Intersection over Union (mIoU), overall Intersection over Union (oIoU), and Precision at threshold X (Pr@X).

mIoU computes the average IoU across all test samples:

(34) $$\mathrm{mIoU}=\frac{1}{M}\sum_{t=1}^{M}\frac{I_{t}}{U_{t}},$$

where *t* is the index of the image–expression–mask triplet, *M* is the total number of test samples, and $I_{t}$ and $U_{t}$ denote the intersection and union areas between the predicted and ground-truth masks, respectively.

oIoU computes the IoU over the aggregated intersection and union across all samples:

(35) $$\mathrm{oIoU}=\frac{\sum_{t=1}^{M}I_{t}}{\sum_{t=1}^{M}U_{t}}.$$

Pr@X measures the percentage of test samples for which the IoU between the predicted and ground-truth masks exceeds a threshold X∈{0.5,0.6,0.7,0.8,0.9}. This metric reflects the localization accuracy at a specific IoU threshold. Higher Pr@X indicates better performance in precisely segmenting target objects.

### 4.3. Implementation Details

We implement SRGFormer in PyTorch 1.7.1. Following prior RRSIS studies [17,32], we adopt Swin-Base pre-trained on ImageNet-22K [45] as the visual encoder and BERT-base-uncased [25] as the textual encoder. All images are resized to 480×480 during both training and testing. Models are optimized using AdamW [46] with a weight decay of 0.01 and an initial learning rate of $3\times 10^{-5}$, which decays according to a polynomial schedule with power 0.9. Training is conducted on two NVIDIA A30 GPUs with Distributed Data Parallel (DDP); the per-GPU batch size is set to 2 (global batch size 4), with 8 data-loading workers per process. Following the training protocols adopted in prior RRSIS studies, we train for 40 epochs on RRSIS-D as in RMSIN [17] and CADFormer [32], and for 60 epochs on RefSegRS as in LGCE [16].

For the graph-modulated progressive decoder, the number of semantic queries is Q=8, the PMR depth is L=3, and the PMR decoder uses 8 attention heads. SRGT contains 2 graph-attention layers with 4 heads, a hidden dimension of 192, dilation rates {1,1,2,4}, and an enabled global sink node. The residual scale of SRGT is initialized as λ=0.1. Deep supervision is applied to all decoder stages with weights [0.3,0.3,0.5,1.0], and the boundary loss weight is set to $\lambda_{bd}=0.5$ with a soft-boundary kernel size of 3. The segmentation loss combines cross-entropy and Dice terms with β=0.1.

### 4.4. Training Convergence Analysis

**Figure 6:**
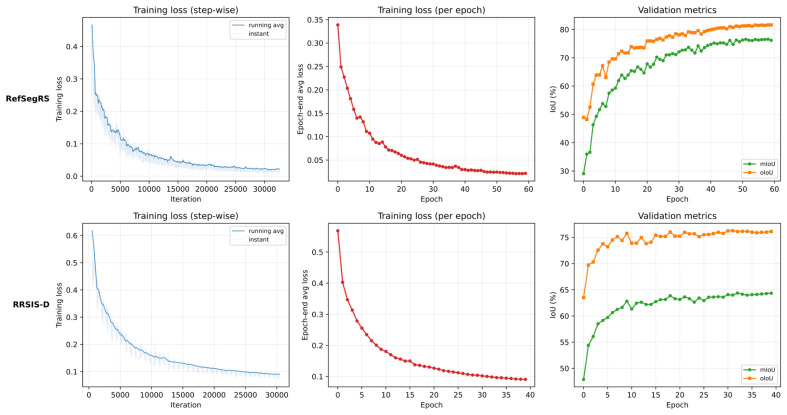
Training convergence curves of SRGFormer on RefSegRS and RRSIS-D.

To further evaluate the training stability of the proposed SRGFormer, we visualize the training loss and validation performance curves on RefSegRS and RRSIS-D, as shown in Figure 6. For both datasets, the step-wise training loss decreases rapidly in the early stage and then gradually converges to a stable range, indicating that the proposed framework can be optimized effectively under the adopted training protocol. The epoch-wise loss curves show a consistent downward trend without abnormal oscillation, further demonstrating stable convergence.

In addition, the validation mIoU and oIoU curves steadily increase as training proceeds and gradually become saturated in the later epochs. On RefSegRS, the validation performance continues to improve until around the last training stage, while on RRSIS-D, the curves converge relatively earlier due to the larger training set and more stable data distribution. These observations suggest that SRGFormer achieves reliable optimization behavior on both benchmarks, and the proposed SRD, SRGT, and PMR modules do not introduce training instability despite the additional semantic decomposition and graph reasoning operations.

### 4.5. Comparison with State-of-the-Art Methods

#### 4.5.1. Compared Methods

To comprehensively evaluate the effectiveness of the proposed SRGFormer, extensive comparisons are conducted against both classic and state-of-the-art approaches in Referring Image Segmentation (RIS) and Remote Sensing Referring Image Segmentation (RRSIS). The competing methods include RRN [40], CMSA [42], LSCM [38], CMPC [30], BRINet [43], CMPC+ [44], LAVT [27], LGCE * [16], FIANet * [31], RMSIN * [17], and CADFormer * [32]. Notably, LGCE *, RMSIN *, FIANet *, and CADFormer * denote the results reproduced by ourselves, whereas the remaining methods are directly reported from the original papers or reproduced by other works.

#### 4.5.2. Results on RefSegRS

Table 2 presents the quantitative comparison on the RefSegRS test set. Notably, SRGFormer adopts the same Swin–BERT multi-modal encoder and backbone fusion strategy as CADFormer [32], enabling a fair evaluation of decoder effectiveness. Unlike CADFormer, which relies on holistic language representations and convolution-dominated decoding, the proposed method explicitly decomposes the referring expression into target, relation, and position semantics, and further incorporates relation-aware graph reasoning and progressive mask refinement during decoding.

Notably, SRGFormer and CADFormer share the identical encoder; all gains are purely decoder-side. As a result, SRGFormer achieves consistent improvements across all evaluation metrics. Specifically, the proposed method improves mIoU from 62.12% to 66.08% and oIoU from 74.10% to 76.93%, demonstrating the effectiveness of structured semantic reasoning for accurate instance segmentation. More importantly, the advantage becomes increasingly evident under stricter evaluation criteria. Compared with CADFormer, SRGFormer achieves gains of 7.72%, 11.28%, and 15.95% at Pr@0.5, Pr@0.6, and Pr@0.7, respectively, indicating substantially stronger instance discrimination capability. Even at the highly challenging Pr@0.9 threshold, where precise boundary localization is required, the proposed method still maintains a performance advantage of 1.77%.

These improvements suggest that explicitly modeling semantic roles and inter-object relations enables the decoder to distinguish among densely distributed same-category instances more effectively than holistic language conditioning. Furthermore, the progressive refinement mechanism continuously injects semantic guidance throughout the decoding process, leading to more accurate object localization and boundary delineation in complex remote sensing scenes.

Compared with earlier methods, SRGFormer also achieves the best overall performance, surpassing FIANet by 0.55% in mIoU and outperforming RMSIN and LAVT by 10.07% and 13.19%, respectively. These results verify that structured semantic decomposition and relation-aware graph reasoning provide more effective guidance for RRSIS than conventional cross-modal alignment alone.

#### 4.5.3. Results on RRSIS-D

Table 3 presents the quantitative comparison on the larger and more challenging RRSIS-D benchmark. Compared with RefSegRS, RRSIS-D contains substantially more image–expression pairs and exhibits greater diversity in object categories, scene layouts, and referring patterns, providing a more rigorous evaluation of model generalization and instance discrimination capability.

As shown in Table 3, SRGFormer achieves the best overall segmentation performance, obtaining an oIoU of 78.66% and an mIoU of 65.87%. Compared with the strongest competing method, CADFormer, the proposed approach improves oIoU and mIoU by 1.37% and 1.46%, respectively. Furthermore, SRGFormer achieves the highest scores at Pr@0.5, Pr@0.6, Pr@0.7, and Pr@0.9, while remaining highly competitive at Pr@0.8 with a score of 43.95%, only 0.02% lower than the best result. These results indicate that the proposed framework consistently maintains superior segmentation quality across different evaluation criteria.

The performance advantage becomes particularly evident under stricter precision thresholds. SRGFormer attains 56.77% at Pr@0.7 and 24.61% at Pr@0.9, outperforming all previous methods. Such improvements suggest that the model is able not only to identify the correct semantic category but also to localize the intended target instance more accurately in scenes containing densely distributed objects and complex referring expressions. The consistent gains achieved on this large-scale benchmark demonstrate the robustness of the proposed framework and its strong generalization ability across diverse remote sensing scenarios.

#### 4.5.4. Qualitative Results

**Figure 7:**
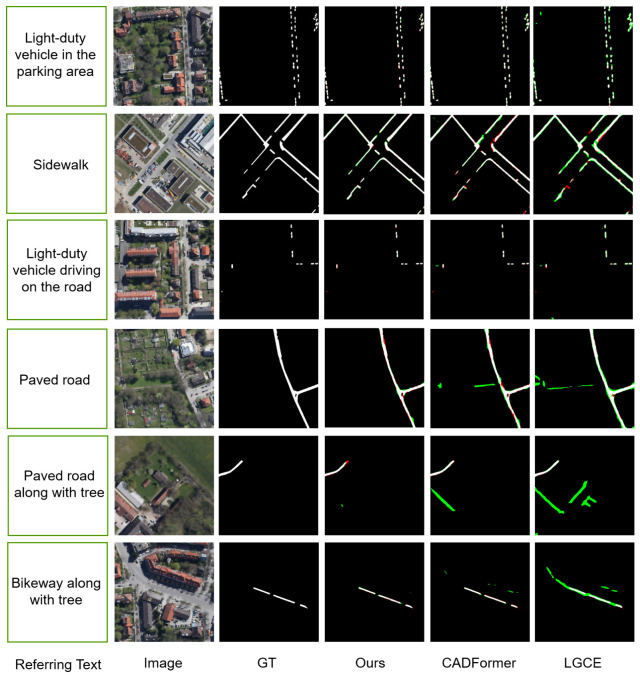
Qualitative comparison of SRGFormer and existing methods on the RefSegRS dataset. White, red, and green regions indicate correct predictions, false negatives, and false positives, respectively.

**Figure 8:**
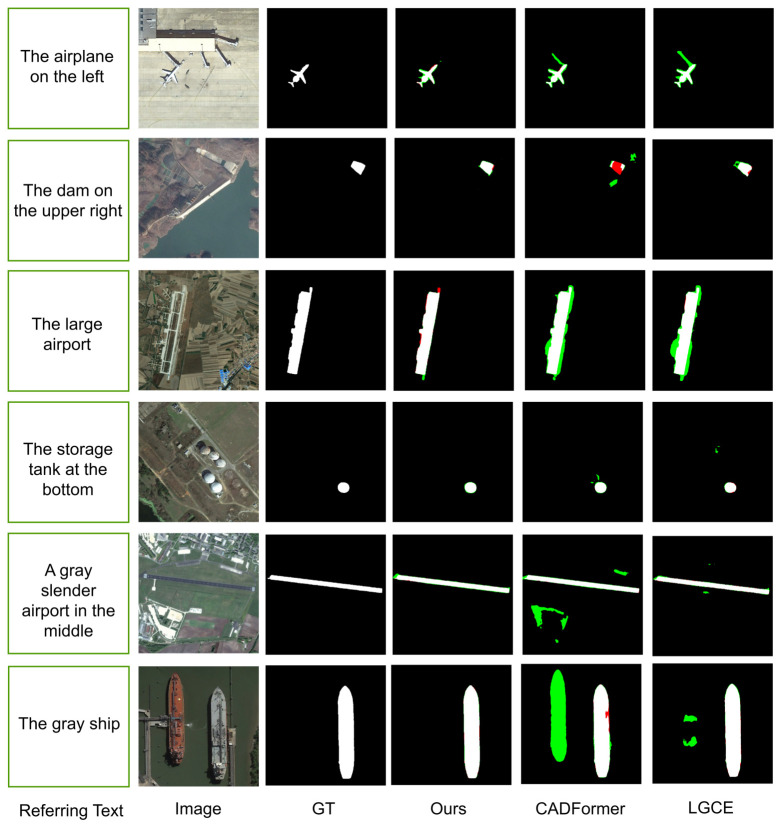
Qualitative comparison of SRGFormer and existing methods on the RRSIS-D dataset. White, red, and green regions indicate correct predictions, false negatives, and false positives, respectively.

To further investigate the behavior of different methods, qualitative comparisons on both RefSegRS and RRSIS-D are presented in Figure 7 and Figure 8, respectively. The selected examples cover a variety of challenging scenarios, including densely distributed same-category objects, positional descriptions, small target localization, elongated structures, and large-scale objects.

On RefSegRS, the primary challenge lies in distinguishing the intended target from multiple visually similar instances and preserving the structural integrity of elongated objects. As illustrated in the vehicle-related examples of Figure 7, existing methods frequently confuse neighboring instances or generate incomplete masks when multiple vehicles appear in close proximity. In contrast, SRGFormer successfully identifies the referred object and produces predictions that closely match the ground truth. Moreover, for elongated targets such as sidewalks, roads, and bikeways shown in the remaining examples, CADFormer and LGCE often suffer from fragmented predictions, discontinuous structures, or false-positive activations. Benefiting from semantic role decomposition and progressive refinement, the proposed method preserves object continuity and achieves more accurate boundary delineation.

The qualitative results on RRSIS-D further demonstrate the robustness of SRGFormer under more diverse object categories and expression patterns. As shown in Figure 8, the proposed method consistently performs better in scenarios requiring precise positional understanding and instance-level discrimination. For example, in the expressions ‘The airplane on the left”, ‘The dam on the upper right”, and ‘The storage tank at the bottom”, competing methods frequently activate irrelevant regions or confuse nearby objects, whereas SRGFormer accurately localizes the target according to the specified spatial cues. Similarly, for scenes containing multiple objects of the same category, such as the ‘gray ship” example, our method effectively suppresses distracting instances and selects the correct target.

Overall, the qualitative comparisons reveal three major advantages of SRGFormer. First, the proposed framework exhibits stronger instance discrimination capability when multiple same-category objects coexist. Second, it demonstrates superior understanding of relational and positional descriptions, leading to more accurate target localization. Third, the generated masks are generally more complete and structurally consistent, particularly for elongated objects and complex boundaries. These observations are consistent with the quantitative results and further verify the effectiveness of explicit semantic role decomposition, relation-aware graph reasoning, and progressive mask refinement for referring remote sensing image segmentation.

### 4.6. Ablation Study

To comprehensively evaluate the effectiveness of the proposed framework, ablation experiments are conducted from two perspectives: (1) the contribution of each core module to the overall performance, and (2) the effectiveness of individual design choices within each module.

For the first aspect, module-level ablations are performed on both the RefSegRS and RRSIS-D test sets to verify the effectiveness and complementarity of the proposed semantic role decomposition (SRD), semantic-relational graph transformer (SRGT), and progressive mask refinement (PMR) modules. All ablation variants are trained under the same experimental settings as the full model to ensure fair comparisons.

For the second aspect, detailed component-level ablations are conducted on the RefSegRS dataset. Since RRSIS-D contains substantially more training samples and requires significantly higher computational cost, performing exhaustive internal ablation experiments on this benchmark would be prohibitively expensive while providing largely consistent conclusions. Therefore, RefSegRS is adopted as the primary benchmark for analyzing the contribution of individual subcomponents and design choices within each module.

#### 4.6.1. Effectiveness of SRD, SRGT, and PMR

Table 4 reports the ablation results of all module combinations on both RefSegRS and RRSIS-D, allowing us to evaluate the individual contribution of each module as well as their interactions.

From the single-module results, all three proposed components consistently improve performance over the baseline on both datasets, demonstrating their individual effectiveness. On RefSegRS, introducing SRD alone increases mIoU from 61.89% to 63.92%, while SRGT and PMR individually improve mIoU to 62.45% and 62.85%, respectively. Similar trends are observed on RRSIS-D, where all three modules yield consistent gains in both mIoU and oIoU. Among the individual components, SRD provides the largest standalone improvement, indicating that explicitly disentangling target, relation, and position semantics offers a stronger linguistic foundation for subsequent reasoning and decoding.

The pairwise combinations further reveal complementary effects among different modules. Combining SRD with PMR improves mIoU to 64.55% on RefSegRS and 64.85% on RRSIS-D, while combining SRD with SRGT achieves even higher scores of 64.83% and 65.21%, respectively. In contrast, the SRGT+PMR combination without SRD produces noticeably smaller gains. This observation suggests that both graph reasoning and progressive refinement benefit substantially from explicit semantic role representations, confirming the importance of SRD as the semantic foundation of the overall framework.

The best performance is consistently achieved when all three modules are jointly enabled. On RefSegRS, the complete model reaches 66.08% mIoU and 76.93% oIoU, representing gains of 4.19% and 4.87% over the baseline, respectively. On RRSIS-D, the full model achieves 65.87% mIoU and 78.66% oIoU, surpassing the baseline by 2.15% and 1.81%. Notably, the performance improvement of the full model is larger than that obtained by any individual module or pairwise combination, indicating a clear synergistic effect among SRD, SRGT, and PMR.

#### 4.6.2. Analysis of SRD

Table 5 analyzes the contribution of different semantic branches in SRD on the RefSegRS test set. Since target semantics provide the fundamental category-level guidance for referring segmentation, we treat the Target branch as the basic component and progressively introduce Relation and Position semantics. Using only the Target branch achieves 63.42% mIoU, indicating that explicit object-level semantics already provide useful guidance for mask prediction. When Relation semantics are further incorporated, the mIoU increases to 65.12%, demonstrating that relational cues are important for distinguishing the referred instance from surrounding objects with similar appearances. Adding Position semantics also improves the performance to 64.58% mIoU, confirming the benefit of spatial cues for target localization.

The complete SRD module, which jointly exploits Target, Relation, and Position semantics, achieves the best performance across all metrics, with 82.68% Pr@0.5, 57.95% Pr@0.7, 76.93% oIoU, and 66.08% mIoU. These results demonstrate that the three semantic roles are complementary: Target semantics determine what object should be segmented, Relation semantics help identify which instance is referred to, and Position semantics provide where-oriented spatial constraints. Their joint modeling enables more accurate semantic guidance for complex referring expressions in remote sensing scenes.

**Figure 9:**
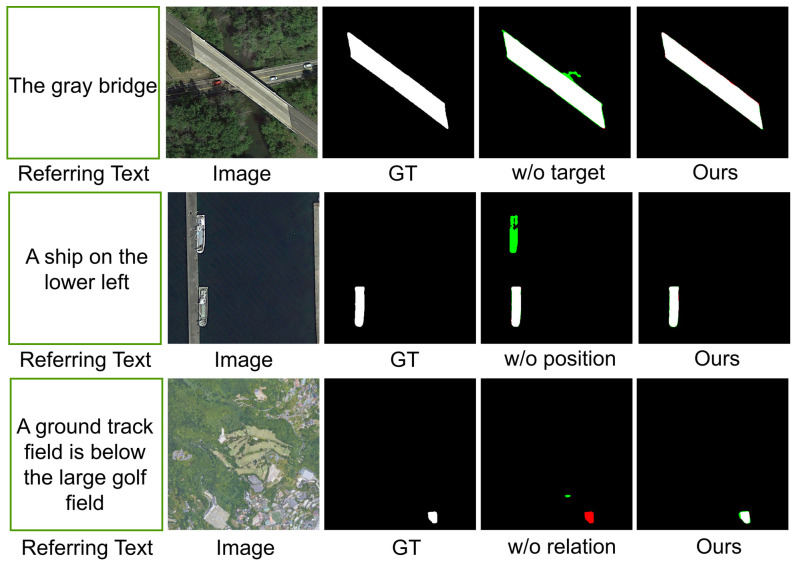
Qualitative analysis of different semantic branches in SRD. Removing $z_{t}$, $z_{p}$, or $z_{r}$ leads to different failure patterns, while the complete SRGFormer achieves more accurate target segmentation.

To further clarify the branch-specific functions of SRD, we perform qualitative ablation analysis, as illustrated in Figure 9. Separate removal of each semantic branch triggers disparate failure cases. Specifically, target semantics govern category-level activation of the referred object; position semantics impose spatial constraints to guarantee localization precision; relation semantics distinguish target instances from visually analogous distractors under relative spatial contexts. Consistent with the quantitative results in Table 5, the three branches offer complementary semantic guidance during decoding, specializing in object recognition, instance discrimination, and spatial localization, respectively.

#### 4.6.3. Analysis of SRGT

To better understand the contribution of different design choices in SRGT, we conduct two groups of ablation experiments on the RefSegRS test set. The first group evaluates the effectiveness of the major components within SRGT, including the graph reasoning mechanism, multi-scale neighborhood modeling, and relation-aware modulation. The second group investigates the impact of different graph neighborhood configurations by varying the dilation rates used for graph construction.

Table 6 presents the component-wise ablation results. The ‘Graph” variant employs only basic graph message passing on visual features without multi-scale neighborhoods or relation-aware modulation. ‘Multi-scale” introduces multi-scale graph construction to capture object interactions at different spatial ranges, while “Relation-aware” further incorporates the relational semantic representation $z_{r}$ to dynamically modulate graph attention. The complete SRGT integrates all three components.

As shown in Table 6, the basic graph reasoning module already improves feature interaction among spatially separated regions, achieving 63.85% mIoU. Introducing multi-scale neighborhood modeling further increases the performance to 64.95% mIoU, indicating that object relations in remote sensing imagery exist across multiple spatial ranges. Incorporating relation-aware modulation also yields a noticeable gain, demonstrating the benefit of explicitly injecting relational semantics into graph reasoning. When all three components are jointly enabled, the complete SRGT achieves the best performance of 66.08% mIoU and 76.93% oIoU, confirming that multi-scale structural reasoning and semantic relation guidance provide complementary benefits for instance discrimination.

To further analyze the impact of different neighborhood dilation configurations on reasoning performance, Table 7 compares different dilation-rate settings. Here, a dilation rate of *d* indicates that each graph node interacts with neighboring nodes sampled at spatial interval *d*, thereby controlling the receptive field of graph message passing.

As shown in Table 7, enlarging the dilation set consistently improves segmentation quality. Using only a single dilation rate of 1 achieves 64.95% mIoU, while expanding the receptive field to {1,2} and {1,2,4} further increases mIoU to 65.36% and 65.82%, respectively. The best performance is obtained with {1,1,2,4}, reaching 66.08% mIoU and 76.93% oIoU, which indicates that repeating a short-range dilation and combining it with longer-range neighbors provides a more effective balance between local context and long-range relational reasoning. When the largest dilation is extended to 8, the performance slightly drops to 65.91% mIoU, suggesting that an excessively large receptive field may introduce noisy interactions and weaken relation-specific message passing.

#### 4.6.4. Analysis of PMR

To further investigate the effectiveness of the proposed progressive mask refinement (PMR) module, we conduct two groups of ablation experiments on the RefSegRS test set. The first group evaluates the contribution of different PMR components, including Semantic Prior Modulation (SPM), Semantic-Specialized Query Initialization (SQI), and Progressive Refinement (PR). The second group analyzes the influence of refinement depth by varying the number of refinement stages *L*.

Table 8 presents the component-wise ablation results. Among all partial configurations, SPM alone achieves 64.02% mIoU, indicating that explicitly injecting target, relation, and position semantics into visual features already provides effective guidance for mask prediction. Introducing SQI further improves mIoU to 65.08%, demonstrating that semantically initialized queries facilitate more effective interaction between linguistic semantics and visual representations. Similarly, combining SPM with PR achieves 64.85% mIoU, confirming the effectiveness of iterative mask refinement under persistent semantic guidance.

When all three components are jointly enabled, the complete PMR achieves the best performance across all evaluation metrics, reaching 66.08% mIoU and 76.93% oIoU. Compared with the SPM-only variant, the full PMR improves mIoU by 2.06%, highlighting the complementary roles of semantic modulation, semantic-aware query initialization, and progressive refinement in translating high-level semantic reasoning into accurate pixel-level segmentation masks.

To further analyze the refinement strategy, Table 9 compares different refinement depths. As the number of refinement stages increases from L=1 to L=3, the segmentation performance consistently improves, with mIoU increasing from 64.92% to 66.08%. This trend demonstrates that progressively refining mask predictions under continuous semantic guidance effectively enhances target localization and boundary quality. The best performance is achieved at L=3, suggesting that three refinement stages provide a favorable balance between semantic correction and spatial detail recovery. Although increasing the depth to L=4 still produces competitive results, a slight decrease in mIoU and oIoU is observed, indicating that excessive refinement may introduce redundant corrections and diminish the benefits of iterative decoding.

Overall, the results verify that PMR effectively bridges the gap between high-level semantic reasoning and pixel-level mask generation. Both semantic-aware query design and progressive refinement contribute substantially to the final performance, while an appropriate refinement depth is crucial for achieving optimal segmentation accuracy.

### 4.7. Discussion and Analysis

#### 4.7.1. Computational Complexity Analysis

Table 10 compares the computational complexity of different methods in terms of FLOPs, parameter number, and inference time. To ensure a fair comparison, all inference time measurements are conducted under the same hardware environment and experimental settings, including the same GPU device, input resolution, and batch size. Compared with existing RRSIS methods, SRGFormer introduces additional semantic role decomposition, relation-aware graph reasoning, and progressive mask refinement operations. As a result, the proposed model requires 641.12G FLOPs, which is higher than the compared methods. This increase mainly comes from the graph-based reasoning process and iterative refinement operations, which are designed to model structured semantic relations and improve instance-level localization.

Although SRGFormer has higher theoretical FLOPs, its parameter size remains moderate. Specifically, the proposed model contains 182.20M parameters, which is considerably smaller than RMSIN, FIANet, and CADFormer. Compared with CADFormer, SRGFormer reduces the parameter number from 272.39M to 182.20M while achieving better segmentation accuracy. This indicates that the performance improvement of SRGFormer is not obtained by simply increasing model capacity, but by introducing more effective semantic decomposition and relation-aware reasoning mechanisms.

In terms of practical inference efficiency, SRGFormer requires 0.1685 s per image. Although it is slower than CADFormer due to the additional graph reasoning and progressive refinement stages, the runtime remains within an acceptable range for referring remote sensing image segmentation. Considering the consistent improvements in mIoU, oIoU, and high-threshold precision, SRGFormer achieves a reasonable trade-off between segmentation accuracy and computational cost.

#### 4.7.2. Failure Case Analysis

**Figure 10:**
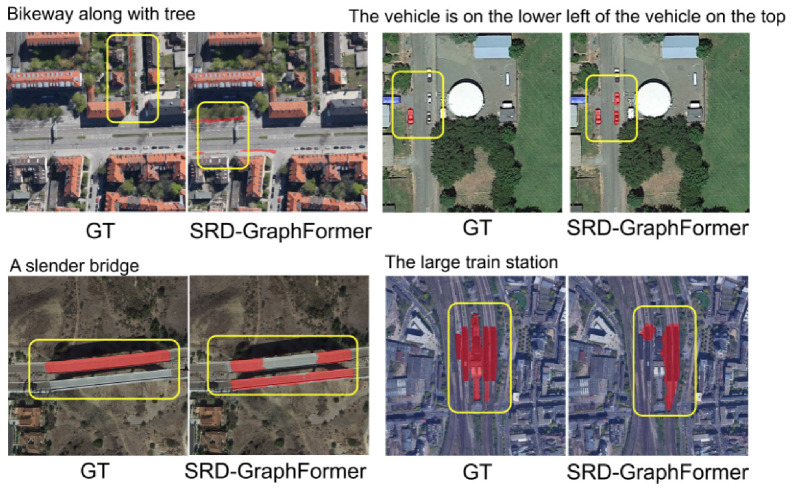
Failure cases of SRGFormer on challenging remote sensing referring segmentation examples. Yellow boxes indicate regions with prediction errors.

Although SRGFormer achieves competitive performance on both RefSegRS and RRSIS-D, several challenging cases still remain, as shown in Figure 10. These failures also reveal the limitations of the proposed modules under extreme scenarios.

First, elongated objects such as bikeways and slender bridges are still difficult to segment accurately. As shown in the highlighted cases of “bikeway along with tree” and “A slender bridge” in Figure 10, the predicted masks may suffer from discontinuity or confusion with nearby linear structures. This is mainly because such targets usually occupy narrow regions and have weak visual contrast with surrounding roads, shadows, or vegetation. Although SRGT enhances long-range relational reasoning among visual regions, it mainly focuses on adaptive feature interaction and cannot fully recover fine spatial details lost during hierarchical feature extraction. Therefore, preserving high-resolution structural information and introducing boundary-aware refinement strategies remain important directions for improving thin-object segmentation.

Second, complex relational expressions involving multiple small objects may still lead to localization ambiguity. For example, in the expression “The vehicle is on the lower left of the vehicle on the top” shown in Figure 10, the model is required to distinguish several visually similar vehicles and infer their relative spatial relationship. Although SRGFormer introduces SRD and SRGT to provide relation-guided reasoning, the semantic representations are learned only through segmentation supervision without explicit semantic-role annotations. Consequently, when multiple candidate objects share similar appearances and relational contexts, the learned relation guidance may still be insufficient to completely resolve the ambiguity. Future work could explore stronger language supervision or uncertainty-aware semantic reasoning to improve robustness for complex expressions.

Third, large-scale functional regions remain challenging due to ambiguous semantic boundaries. In the case of “The large train station” in Figure 10, the target covers a complex region composed of platforms, tracks, and surrounding facilities. Since the boundary of such a semantic region is not always visually clear, PMR may refine the mask based on available semantic and visual representations but still struggle with uncertain peripheral regions. Incorporating explicit boundary uncertainty modeling and region-level reasoning may further improve segmentation consistency for large functional objects.

Overall, these failure cases indicate that while SRD, SRGT, and PMR effectively improve semantic guidance, relational reasoning, and progressive mask refinement, they still face challenges in recovering extremely fine structures, resolving highly ambiguous language descriptions, and modeling uncertain semantic boundaries. These observations motivate future research toward high-resolution representation learning, stronger language grounding, and uncertainty-aware segmentation strategies.

## 5. Conclusions

In this paper, we proposed SRGFormer, a semantic role decomposition and graph reasoning framework for referring remote sensing image segmentation. Existing RRSIS methods usually represent the entire referring expression as a holistic language embedding and rely on convolution-dominated decoding, which limits their ability to distinguish among densely distributed same-category objects and handle complex expressions involving target categories, inter-object relations, and spatial positions. To address this limitation, SRGFormer explicitly decomposes the referring expression into target, relational, and positional semantics, enabling more structured linguistic guidance for downstream segmentation.

Based on the decomposed semantic roles, we further designed a semantic-relational graph transformer to perform relation-aware graph reasoning over multi-scale visual features. This allows the model to capture long-range dependencies among candidate objects and improves instance-level discrimination in cluttered remote sensing scenes. In addition, a progressive mask refinement module was introduced to continuously inject semantic priors during the decoding process, thereby alleviating semantic fading and improving pixel-level mask prediction.

Extensive experiments on the RefSegRS and RRSIS-D datasets demonstrate that SRGFormer achieves competitive performance compared with existing state-of-the-art methods. The ablation studies further verify the effectiveness of the proposed SRD, SRGT, and PMR modules, as well as the necessity of semantic branch decomposition, graph neighborhood design, and progressive refinement depth. Qualitative results also show that the proposed method produces more accurate and structurally consistent masks in challenging scenarios involving small objects, elongated structures, positional descriptions, and densely distributed same-category instances.

Despite these improvements, SRGFormer still has several limitations. Extremely small objects, ambiguous referring expressions, and large functional regions with unclear semantic boundaries remain challenging. In future work, we will explore higher-resolution feature preservation, uncertainty-aware language grounding, and more explicit boundary modeling to further improve the robustness and generalization ability of referring remote sensing image segmentation.

## Figures and Tables

**Table 1:** Statistics of the two RRSIS datasets.

Dataset	Total Triplets	Scenes	Categories	Train/Val/Test
RefSegRS	4420	285	14	2172/431/1817
RRSIS-D	17,402	–	20	12,181/1740/3481

**Table 2:** Quantitative comparisons with state-of-the-art RRSIS methods on RefSegRS. The best results are highlighted in red, the second-best results are marked in green, and the third-best results are marked in blue. * denotes results reproduced by ourselves.

Methods	Pub	Vision	Language	Pr@5	Pr@6	Pr@7	Pr@8	Pr@9	oIoU	mIoU
BRINet [43]	CVPR20	R-101	LSTM	20.72	14.26	9.87	2.98	1.14	58.22	31.51
LSCM [38]	ECCV20	R-101	LSTM	31.54	20.41	9.51	5.29	0.84	61.27	35.54
CMPC [30]	CVPR20	R-101	LSTM	32.36	14.14	6.55	1.76	0.22	55.39	40.63
CMSA [42]	CVPR19	R-101	None	28.07	20.25	12.71	5.61	0.83	64.53	41.47
RRN [40]	CVPR18	R-101	LSTM	30.26	23.01	14.87	7.17	0.98	65.06	41.88
CMPC+ [44]	TPAMI21	R-101	LSTM	49.19	28.31	15.31	8.12	2.55	66.53	43.65
LAVT [27]	CVPR22	Swin-B	BERT	63.37	48.73	27.17	12.71	3.69	75.04	52.89
LGCE * [16]	TGRS24	Swin-B	BERT	73.60	61.08	39.34	15.96	5.42	76.66	59.90
RMSIN * [17]	CVPR24	Swin-B	BERT	68.24	53.77	29.11	10.95	1.82	71.04	56.01
FIANet * [31]	TGRS25	Swin-B	BERT	81.51	72.10	53.49	24.99	4.57	76.47	65.53
CADFormer * [32]	JSTARS25	Swin-B	BERT	74.96	61.70	42.00	18.35	3.35	74.10	62.12
SRGFormer	Ours	Swin-B	BERT	**82.68**	**72.98**	**57.95**	**26.75**	**5.12**	**76.93**	**66.08**

**Table 3:** Quantitative comparisons with state-of-the-art RRSIS methods on RRSIS-D. The best results are highlighted in red, the second-best results are marked in green, and the third-best results are marked in blue. * denotes results reproduced by ourselves.

Methods	Pub	Vision	Language	Pr@5	Pr@6	Pr@7	Pr@8	Pr@9	oIoU	mIoU
RRN [40]	CVPR18	R-101	LSTM	51.09	42.47	33.04	20.80	6.14	66.53	46.06
CMSA [42]	CVPR19	R-101	None	55.68	48.04	38.27	26.55	9.02	69.68	48.85
LSCM [38]	ECCV20	R-101	LSTM	57.12	48.04	37.87	26.37	7.93	69.28	50.36
CMPC [30]	CVPR20	R-101	LSTM	57.93	48.85	38.50	25.28	9.31	70.15	50.41
LGCE * [16]	TGRS24	Swin-B	BERT	68.10	60.52	52.24	42.24	23.85	76.68	60.16
LAVT [27]	CVPR22	Swin-B	BERT	69.54	63.51	53.16	43.97	24.25	77.59	61.46
FIANet * [31]	TGRS25	Swin-B	BERT	74.26	66.96	55.64	42.09	23.30	76.73	63.90
RMSIN * [17]	CVPR24	Swin-B	BERT	73.17	66.19	55.10	41.86	23.36	77.52	63.44
CADFormer * [32]	JSTARS25	Swin-B	BERT	74.63	67.45	55.82	41.83	23.36	77.29	64.41
SRGFormer	Ours	Swin-B	BERT	**75.70**	**68.40**	**56.77**	**43.95**	**24.61**	**78.66**	**65.87**

**Table 4:** Ablation study on the effectiveness of core modules on the RefSegRS and RRSIS-D test sets. All eight combinations are reported to verify individual contributions and pairwise synergy. Bold values indicate the best performance.

Modules			RefSegRS					RRSIS-D				
SRD	SRGT	PMR	Pr@0.5	Pr@0.7	Pr@0.9	oIoU	mIoU	Pr@0.5	Pr@0.7	Pr@0.9	oIoU	mIoU
–	–	–	73.58	40.55	2.71	72.06	61.89	73.33	54.26	22.89	76.85	63.72
✓	–	–	75.06	42.37	3.18	73.04	63.92	74.58	54.91	23.34	77.22	64.18
–	✓	–	74.12	41.25	2.95	72.48	62.45	73.85	54.62	23.05	77.05	64.15
–	–	✓	74.45	41.68	3.05	72.75	62.85	74.12	54.78	23.18	77.15	64.35
✓	✓	–	79.84	51.86	4.46	75.12	64.83	75.08	55.82	24.03	78.05	65.21
✓	–	✓	76.35	43.65	3.82	73.85	64.55	74.95	55.45	23.72	77.68	64.85
–	✓	✓	75.28	42.15	3.45	73.25	63.95	74.68	55.25	23.55	77.52	65.05
✓	✓	✓	**82.68**	**57.95**	**5.12**	**76.93**	**66.08**	**75.70**	**56.77**	**24.61**	**78.66**	**65.87**

**Table 5:** Ablation study on semantic branches of SRD on the RefSegRS test set. Bold values indicate the best performance.

Target	Relation	Position	Pr@0.5	Pr@0.7	Pr@0.9	oIoU	mIoU
✓	–	–	78.26	50.48	4.37	74.38	63.42
✓	✓	–	80.45	54.82	4.95	75.68	65.12
✓	–	✓	79.86	53.45	4.88	75.25	64.58
✓	✓	✓	**82.68**	**57.95**	**5.12**	**76.93**	**66.08**

**Table 6:** Component-wise ablation study of SRGT on the RefSegRS test set. Bold values indicate the best performance.

Graph	Multi-Scale	Relation-Aware	Pr@0.5	Pr@0.7	Pr@0.9	oIoU	mIoU
✓	–	–	78.52	50.36	4.35	74.68	63.85
✓	✓	–	80.21	53.88	4.82	75.72	64.95
✓	–	✓	79.36	52.15	4.68	75.15	64.52
✓	✓	✓	**82.68**	**57.95**	**5.12**	**76.93**	**66.08**

**Table 7:** Sensitivity analysis of different dilation-rate configurations in SRGT on the RefSegRS test set. Bold values indicate the best performance.

Dilation Rates	Pr@0.5	Pr@0.7	Pr@0.9	oIoU	mIoU
{1}	80.21	53.88	4.82	75.72	64.95
{1,2}	81.12	55.18	4.95	76.15	65.36
{1,2,4}	82.03	56.42	5.03	76.58	65.82
{1,1,2,4}	**82.68**	**57.95**	**5.12**	**76.93**	**66.08**
{1,2,4,8}	82.18	56.95	5.05	76.63	65.91

**Table 8:** Ablation study of PMR components on the RefSegRS test set. Bold values indicate the best performance.

SPM	SQI	PR	Pr@0.5	Pr@0.7	Pr@0.9	oIoU	mIoU
✓	–	–	79.85	52.48	4.56	74.92	64.02
✓	✓	–	81.05	54.92	4.81	75.88	65.08
✓	–	✓	80.72	54.35	4.75	75.62	64.85
✓	✓	✓	**82.68**	**57.95**	**5.12**	**76.93**	**66.08**

**Table 9:** Ablation study on the refinement depth of PMR on the RefSegRS test set. Bold values indicate the best performance.

PMR Depth *L*	Pr@0.5	Pr@0.7	Pr@0.9	oIoU	mIoU
1	80.52	54.86	4.88	75.42	64.92
2	81.65	56.12	4.97	76.05	65.56
3	**82.68**	**57.95**	5.12	**76.93**	**66.08**
4	82.41	57.36	**5.15**	76.72	65.92

**Table 10:** Computational complexity comparison of different methods. Bold values indicate the best performance.

Methods	FLOPs ↓	Params ↓	Inference Time ↓
LAVT [27]	**383.84G**	**118.85M**	**0.0878 s**
LGCE [16]	401.88G	167.37M	0.0930 s
RMSIN [17]	433.02G	240.04M	0.1264 s
FIANet [31]	435.87G	256.17M	0.1365 s
CADFormer [32]	468.14G	272.39M	0.1446 s
SRGFormer (Ours)	641.12G	182.20M	0.1685 s

## Data Availability

Experiments in this work are conducted on two datasets, RefSegRS and RRSIS-D. The RefSegRS dataset is publicly available at https://github.com/zhu-xlab/rrsis, accessed on 9 June 2026. The RRSIS-D dataset can be obtained at https://github.com/Lsan2401/RMSIN, accessed on 9 June 2026. All additional data and source code are available upon reasonable request from the corresponding author.

## Abbreviations

The following abbreviations are used in this manuscript:

Acronym	Definition
BERT	Bidirectional Encoder Representations from Transformers
CNN	Convolutional Neural Network
GAT	Graph Attention Network
IoU	Intersection over Union
mIoU	mean Intersection over Union
oIoU	overall Intersection over Union
PMR	Progressive Mask Refinement
RIS	Referring Image Segmentation
RRSIS	Referring Remote Sensing Image Segmentation
SMA	Stage-wise Mutual Alignment
SMGAM	Semantic Mutual Guidance Alignment Module
SRD	Semantic Role Decomposition
SRGFormer	Semantic Role-Guided Graph Reasoning Framework
SRGT	Semantic-Relational Graph Transformer
